# Correlation of Viral Load With the Clinical and Biochemical Profiles of COVID-19 Patients

**DOI:** 10.7759/cureus.16655

**Published:** 2021-07-27

**Authors:** Muhammad Atique, Atif Ghafoor, Rabia Javed, Noor Fatima, Anam Yousaf, Samana Zahra

**Affiliations:** 1 Histopathology, Pakistan Kidney and Liver Institute & Research Center, Lahore, PAK; 2 Molecular Biology, Pakistan Kidney and Liver Institute & Research Center, Lahore, PAK

**Keywords:** covid-19, cycle threshold, clinical severity, viral load, rt-pcr, sars-cov-2

## Abstract

Background/objective

Coronavirus infectious disease (COVID-19) is a novel disease caused by severe acute respiratory syndrome coronavirus 2 (SARS-COV-2). Some studies have shown that disease severity according to clinical and biochemical parameters are in direct relation to viral load while others have found no direct correlation. In this study, the COVID-19 cycle threshold (Ct) value, which is taken as a direct indicator of the viral load, has been correlated with the biochemical and clinical parameters in COVID-19 patients.

Methods

In this cross-sectional, retrospective, and single-center study, 365 patients admitted with COVID 19 were divided into three groups according to their Ct values obtained from reverse transcription-polymerase chain reaction RT-PCR as 1 (9-20), 2 (21-30), and 3 (31-40). The correlation of the COVID-19 Ct value with biochemical parameters and clinical presentation (taken as mild, moderate, and severe) was done and analyzed. The chi-square test was used for the correlation and calculated by using SPSS V-24.0 (IBM Corp., Armonk, NY). p-value <0.05 was considered significant statistically.

Results

Disease severity levels (mild, moderate, and severe) correlated in group 1 (Ct value 9 to 20), 2 (Ct value 21 to 30), and 3 (Ct value 31 to 40) but no significance was found between disease severity levels and the Ct value groups' p-value (>0.05). All the biochemical parameters analyzed (alanine transaminase (ALT), aspartate aminotransferase (AST), albumin, bilirubin, c-reactive protein (CRP), lactate dehydrogenase (LDH), ferritin, D-dimer, and total leucocyte count (TLC)) showed a significant p-value (<0.05) in all the three groups studied. Procalcitonin (PCT), however, did not show any significant value in any of the groups studied.

In the intergroup assessment, it was found that the values of ALT, AST, albumin, CRP, ferritin, bilirubin, and TLC are maximum in group 2 with a downward trend in groups 1 and 2. Neutrophils and lymphocytes did not show any variations. LDH did not follow the trend of increasing viral load.

Conclusions

The severity of the disease was not statistically significant in the Ct value groups (p> 0.05). However biochemical parameters, i.e. ALT, AST, ALP, CRP, and bilirubin were statistically significant (p<0.05).

Patients with COVID-19 should be closely monitored for the assessment of disease progression according to the above-mentioned biochemical parameters.

## Introduction

The year 2019 was the year of the coronavirus disease (COVID-19) pandemic. It is caused by the severe acute respiratory syndrome coronavirus 2 (SARS-CoV-2). Although this virus was first noted in Wuhan, China, in December 2019, it has by now spread dramatically to 209 countries of the world, which includes Pakistan. The first case of this virus was reported in Karachi, Pakistan, on February 26, 2020 [1]. By June 17, 2020, every district of Pakistan had reported at least one case of coronavirus. Pakistan is at the tenth position among Asian countries with the highest number of COVID-19 cases and in the 23rd position amongst all countries in the world with respect to the highest number of COVID-19 cases [2]. The number of cases increased and reached its peak on June 14, 2020, when 6825 cases were reported on that day alone. Since then, the general trend (with a few peaks and troughs) for COVID-19 positive cases has been on the decline in all provinces of Pakistan. As of July 7, 2021, the total number of cases seen in Pakistan is 966,007, with the total number of deaths being 22,469. The highest number of confirmed cases have been seen in the province of Punjab (n=347,180) followed by Sind (n=342,228) [2].

The virus causing the disease belongs to the B lineage of beta coronavirus of the family Coronaviridae. The coronavirus is named because of its shape (crown) as from Latin - as seen under an electron microscope. These have the largest RNA genome among all the RNA viruses. On encoding the viral protein responsible for a variety of symptoms, including respiratory, gastrointestinal, and neurological, human disease is caused by six different species; out of these, 229E, OC43, and NL63 cause a common cold-like illness in immunocompetent individuals while two others caused the SARS-Cov-2 causing the respiratory distress syndrome in China in 2002-2003 and Middle East respiratory syndrome coronavirus (MERS-Cov) in the Middle East in 2012 [3].

The standard procedure for the molecular diagnosis of COVID-19 mainly relies on detecting the RNA of this virus. The molecular method for coronavirus disease 2019 (COVID-19) diagnosis is via real-time reverse transcription-polymerase chain reaction (RT-PCR). The cycle threshold (Ct) values of RT-PCR represent the number of amplification cycles that are needed by the target gene to surpass a certain threshold level. Ct values are inversely proportional to viral load and can be used as an indirect way of quantifying viral RNA copy numbers [4].

According to the WHO guidelines, COVID-19 disease has been clinically divided into Mild (symptomatic patients without hypoxia or viral pneumonia), Moderate (patients with pneumonia but SpO2 >90%), Severe (patients with severe pneumonia and oxygen saturation < 90% on room air), and Critical Disease (which includes acute respiratory distress syndrome (ARDS) and sepsis) [5].

Multiple biochemical parameters are deranged when a patient is infected with coronavirus, with changes in C reactive protein (CRP) levels, lymphopenia, raised aminotransferases, creatinine kinase, and lactate dehydrogenase (LDH) among others [6].

The aim of this study is to investigate the correlation of viral load with clinical and biochemical parameters. Although there are a few international studies on the topic, rare, if any, work has been done in Pakistan, with no such study of Pakistan available on Google Scholar search. This is the first study in Pakistan correlating viral load, which is inversely proportional to Ct value and likely to be directly related to clinical and laboratory parameters. The results of the study will help doctors better understand the dynamics of this novel virus and help clinicians in the management of patients.

## Materials and methods

Study design and sample collection

This cross-sectional, retrospective study was conducted on COVID-19 PCR positive patients (n=365) who had been admitted to Pakistan Kidney and Liver Institute & Research Center (PKLI & RC) from April 6, 2020, to July 26, 2020. PKLI & RC is a tertiary care reference hospital in Lahore, Province of Punjab, Pakistan. It is a designated hospital for kidney and liver transplant patients; however, it was declared a COVID-19 facility by the Government of Punjab, Pakistan, on March 14, 2020, during the COVID-19 pandemic. The study protocol was approved by the Institutional Review Board (IRB) of PKLI & RC.

Sample inclusion criteria

Confirmed PCR-positive patients (patients who have a Ct value ≤40 for COVID-19, with the following criteria, were included in this study: 1. Only nasopharyngeal (NP) swabs were processed for testing; 2. Only properly labeled and barcoded vials with intact sample quality were processed for testing; 3. Biochemical parameters were analyzed on serum samples taken from the COVID-19 PCR-positive patients (n=365); 4. Clinical parameters were analyzed on COVID-19 PCR-positive patients who were admitted to PKLI & RC.

Sample exclusion criteria

The following samples were not included in this study: 1. Suspected Covid-19 patients with PCR-negative reports; 2. Improperly labeled vials and samples with thick mucus; 3. Samples with compromised integrity, e.g., viral transport medium (VTM) or swab missing in the sample vial, leaked VTM vial, hemolyzed samples, and so on; 4. Non-admitted patients.

RT-PCR for COVID-19

Laboratory confirmation of SARS-CoV-2 was performed by RT-PCR at the Molecular Laboratory of Pathology Department, which is a Bio-Safety Level (BSL-II) Laboratory at PKLI & RC.

Viral RNA was isolated from NP swabs in VTM by using the Versant kPCR system (Siemens, Munich, Germany). The amplification and detection of viral RNA were performed on Quant Studio 5DX molecular system (Applied Biosystems, Waltham, Massachusetts). The amplification/detection is based on the principle of RT-PCR, which simultaneously amplifies and detects two genes: 1) Open reading frame 1ab (ORF-1ab) and 2) Nucleocapsid protein (N) genes. The fluorescent channels FAM and ROX were used to detect the ORF-1ab and N genes, respectively, whereas the CY5 channel was used to detect the fluorescent signal of internal control (IC). The RT-PCR detection system also amplifies a known positive internal control with each reaction, which monitors the presence of PCR inhibitors in the test specimens, to avoid a false-negative result.

An RT-PCR assay was performed using the following thermocycler protocol: Reverse transcription at 50^o^C for 15 minutes, cDNA pre-denaturation at 95^o^C for 1 minute, and denaturation at 95^o^C or 15 seconds, annealing, extension, and fluorescence collection at 60^o^C for 30 sec. Denaturation and annealing were repeated for five cycles.

On the basis of Ct values, the patients were divided into three groups (Table 1). 

**Table 1:** Ct values groups Ct: cycle threshold

Group	Ct Values
1	9-20
2	21-30
3	31-40

Clinical categories

1) Mild disease: These included those symptomatic patients who had no viral pneumonia and/or hypoxia.

2) Moderate disease: These included those patients who had an indication of pneumonia, including signs like cough, fever, fast breathing, dyspnea, and oxygen saturation ≥90%.

3) Severe disease: These are the patients having severe pneumonia with oxygen saturation <90% on room air (55-60 mm Hg), as well as ARDS and septicemia.

Laboratory parameters

The patients were tested for the following biochemical laboratory parameters:

1) Chemistry parameters: Total bilirubin, CRP, LDH, albumin, alanine aminotransferase (ALT), aspartate aminotransferase (AST), ferritin, and pro-calcitonin tests were performed on serum samples taken from COVID-19 PCR-positive patients. All biochemical tests were carried out on the automated Alinity module c and module hi (Abbott Diagnostics, Chicago, Illinois).

2) Hematological parameters: Total leucocyte count (TLC), D-dimer, lymphocytes, and neutrophils.

Statistical analysis

Data on clinical and biochemical parameters, including total bilirubin, CRP, LDH, albumin, ALT, AST, ferritin, pro-calcitonin, TLC, D-dimer, lymphocytes, and neutrophils were demonstrated as mean ± standard deviation. The Spearman correlation analysis between disease severity groups and Ct values was calculated using SPSS v24.0 (IBM Corp., Armonk, NY). Two-tailed p <0.05 was considered statistically significant for the current study.

## Results

In this study, a total of 365 patients were included and divided into three groups.

Group 1 (Ct value 9 to 20) (n=60)

In this group, we observed that infection of COVID-19 in male patients (n=40; 66.6%) as compared to female patients (n=20; 33.4%). And all patients in this group have been classified clinically into mild (86.7%), moderate (10%), and severe (3.3%). Whereas blood biochemistry parameters were ALT (60.65 ± 90.08; p-value 0.007), AST (37.0 ± 29.30; p-value 0.0), albumin (4.00 ± 0.58; p-value 0.0), CRP (1.49 ± 3.14; p-value 0.018), bilirubin total (0.67 ± 0.37; p-value 0.0), LDH (313.8 ± 156.3; p-value 0.004), ferritin (894.9 ± 889.5; 0.002), D-dimer (1419.2 ± 2076.2; 0.059, procalcitonin (PCT; 0.39), lymphocytes (2.09 ± 0.72; p-value 0.0), neutrophils (5.13 ± 3.60; p-value 0.0), and TLC (8.66 ± 5.1; p-value 0.0).

Group 2 (Ct value 21 to 30) (n=148)

This group's male patients were 74.3% as compared to female patients (25.7%). Clinically, they were classified into mild (84.5%), moderate (8.6%), and severe (6.9%). Blood biochemistry parameters were ALT (69.35 ± 65.10; p-value 0.0), AST (45.82 ± 35.90; p-value 0.0), albumin (3.89 ± 0.54; p-value 0.0), CRP (2.27 ± 4.51; p-value 0.0), bilirubin total (0.65 ± 0.29; p-value 0.0), LDH (363.4 ± 195.2; p-value 0.0), ferritin (1161.8 ± 1632.8; p-value 0.0), D-dimer (1097.7 ± 2237.9; 0.001), PCT (4.99 ±18.8; p-value 0.290), lymphocytes (2.15±0.91; p-value 0.0), neutrophils (5.51 ± 4.68; p-value 0.0), and TLC (8.82 ± 5.19; p-value 0.0).

Group 3 (CT value 31 to 40) (n=157)

In this group, the males patients were 67.5% as compared to female patients 32.5%. The patients in this group were classified clinically into mild (75.7%), moderate (20.6%), and severe (3.7%). Blood parameters showed ALT (44.76 ± 22.78; p-value 0.0), AST (37.37 ± 19.69; p-value 0.0), albumin (3.77 ± 0.64; p-value 0.0), CRP (1.90 ± 2.74; p-value 0.0), bilirubin total (0.62 ± 0.25; p-value 0.0), LDH (395 ± 204.60; p-value 0.0), ferritin (804.7 ± 821.2; p-value 0.0), D-dimer (972.2 ± 1461.1; 0.0), PCT (4.76 ± 13.14; p-value 0.235), lymphocytes (2.20 ± 0.95; p-value 0.0), neutrophils (5.56 ± 3.82; p-value 0.0); and TLC (8.78 ± 4.12; p-value 0.0).

Table 2 provides the correlation of the clinical and biochemical profiles with Ct values in the three groups.

**Table 2:** Correlation of the clinical and biochemical profiles with Ct values in the three groups (n=365) Ct: cycle threshold; ALT: alanine transaminase; AST: aspartate aminotransferase; CRP: c-reactive protein, LDH: lactate dehydrogenase; TLC: total leucocyte count; PCT: procalcitonin

Group		Group 1	Group 2	Group 3
Ct Value		9 to 20	21 to 30	31 to 40
Mean Ct Value		16.58 ± 2.93	26.47 ± 2.78	34.52 ± 2.19
n (365)		60	148	157
Age		41.68 ± 16.58	43.43 ± 16.24	43.41 ± 15.63
Male		40 (66.6%)	110 (74.3 %)	106 (67.5%)
Female		20 (33.4%)	38 (25.7%)	51 (32.5 %)
Clinical	Mild	26 (86.7%)	49 (84.5 %)	81 (75.7%)
	Moderate	3 (10 %)	5 (8.6%)	22 (20.6 %)
	Severe	1 (3.3%)	4 (6.9%)	4 (3.7 %)
	p-value	0.308	0.298	0.394
Biochemical Profile	ALT	60.65 ± 90.08 (0.007)	69.35 ± 65.10 (0.0)	44.76 ± 22.78 (0.0)
	AST	37.0 ± 29.30(0.0)	45.82 ± 35.90(0.0)	37.37 ± 19.69(0.0)
	Albumin	4.00 ± 0.58(0.0)	3.89±0.54(0.0)	3.77 ± 0.64(0.0)
	CRP	1.49 ± 3.14(0.018)	2.27 ± 4.51(0.0)	1.90 ± 2.74(0.0)
	Bili	0.67 ± 0.37(0.0)	0.65 ± 0.29(0.0)	0.62 ± 0.25(0.0)
	LDH	313.8±156.3(0.004)	363.4 ± 195.2(0.0)	395 ± 204.60(0.0)
	Ferritin	894.9 ± 889.5(0.002)	1161.8 ±1632.8(0.0)	804.7 ± 821.2(0.0)
	PCT	0.39	4.99 ±18.8(0.290)	4.76 ± 13.14(0.235)
	Lympho	2.09 ± 0.72(0.0)	2.15±0.91(0.0)	2.20 ± 0.95(0.0)
	Neutro	5.13 ± 3.60(0.0)	5.51 ± 4.68(0.0)	5.56 ± 3.82(0.0)
	TLC	8.66 ± 5.1(0.0)	8.82 ± 5.19(0.0)	8.78 ± 4.12(0.0)
	D-dimer	1419.2 ± 2076.2(0.059)	1097.7 ± 2237.9(0.001)	972.2 ± 1461.1(0.0)

## Discussion

Although the initial diagnosis of SARS-CoV-2 can be made on a qualitative reporting of the test results as merely positive or negative; however, the reporting of Ct values may also help clinicians for better patient management. The quantitative report results of COVID-19 may provide a guide for infection control, public health, and occupational health decisions [7].

The higher Ct values indicate lower viral loads and vice versa. Ct values and log viral loads may not be directly proportional due to the presence of inhibitory factors in clinical samples.

In the current scenario of the COVID-19 pandemic, the clinical knowledge of the disease is constantly evolving, although limited data is available regarding the correlation of the viral loads with patients' prognoses, including disease progression or mortality.

Clinical presentation

It was observed that the lowest Ct value group (9-20) of patients showed no significant p-value as far as the clinical presentation is concerned. This could be because clinical data was only available for 16 patients out of 29, the sampling site is also critical for the true representation of viral load and disease severity samples from lower respiratory tract specimens that included sputum samples exhibited higher viral loads as compared to nasopharyngeal swabs [8].

It has been shown that the laboratory test result reliability depends on multiple factors. The collection of biological material should be carried out at an active infection stage, allowing complete identification of the pathogen. RT-PCR should preferably be performed between the third and fifth days of symptom onset since the viral load is usually higher during the first week of disease [9].

In this study, only nasopharyngeal swabs were taken, which is the initial site of viral replication, and patients could be in the initial phase prior to symptom development [9].

In the second group (Ct value 21-30), patients, however, showed a significant p-value (0.03). This indicates that the disease progression in these patients has a direct correlation with the viral load.

Of the groups with higher Ct values (31- 0), no significant correlation was found to be present between the viral load and disease severity. The reason could also be the site of samples. The disease progression in these patient groups thereby indicates that they were in the recovery phase, hence the viral loads were lower in these patients.

**Figure 1:**
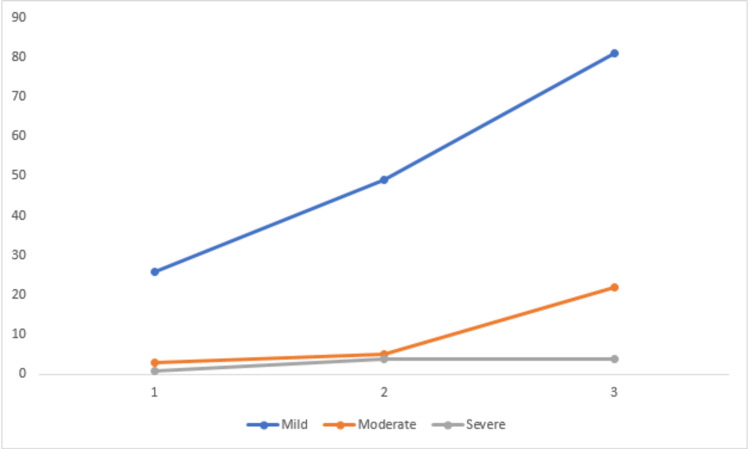
Correlation of clinical parameters with viral load in three groups

There is no clear understanding available as to why most of the infected people show no or mild symptoms of the disease. There is a lack of data regarding the time for the survival or persistence of SARS-CoV-2 viral RNA in the nasopharynx. Also, there is no clear information available for the relationship between viral load as well as the factors that contribute to disease progression and severity in infected individuals (Figure 1) [10].

Biochemical/hematological parameters

Both hematological and biochemical markers have been shown to demonstrate an association with Ct values. Lower Ct values have been shown to be associated with higher lactate dehydrogenase levels. Moreover, the severe COVID-19 cases with lower Ct scores have been shown to have low lymphocyte and T-cells counts; the total and differential neutrophil counts were however observed to be significantly increased [11].

As Ct values were established to associate with a number of biochemical markers. Mardani et al. found that biochemical parameters like ALT, AST, CRP, LDH, bilirubin, and albumin play an important role in the prediction of RT-PCR positive cases for COVID-19. There are increased levels of ALT, AST, and LDH also observed for COVID-19-positive patients. In another study, it was determined that 14%-53% of COVID-19 patients had abnormal levels of ALT and AST, and 2%-11% of cases showed liver comorbidities during the development of COVID-19 disease [12].

Trends of ALT, AST, and Albumin in Different Groups

Alanine transaminase (ALT) and aspartate aminotransferase (AST) are the enzymes that detect liver damage, which has been seen in COVID-19. Zhang et al. found that 2%-11% of COVID-19 patients had liver comorbidities while 14%-53% of the patients had abnormal levels of ALT and AST during disease progression [13-14].

In this study, ALT increased in three groups (1, 2, and 3) and showed a significant p-value (p<0.05). Whereas AST also increased in the three groups and showed a significant p-value (p<0.05) in all three groups. Both parameters have a downward trend from high viral load groups to lower viral load groups.

**Figure 2:**
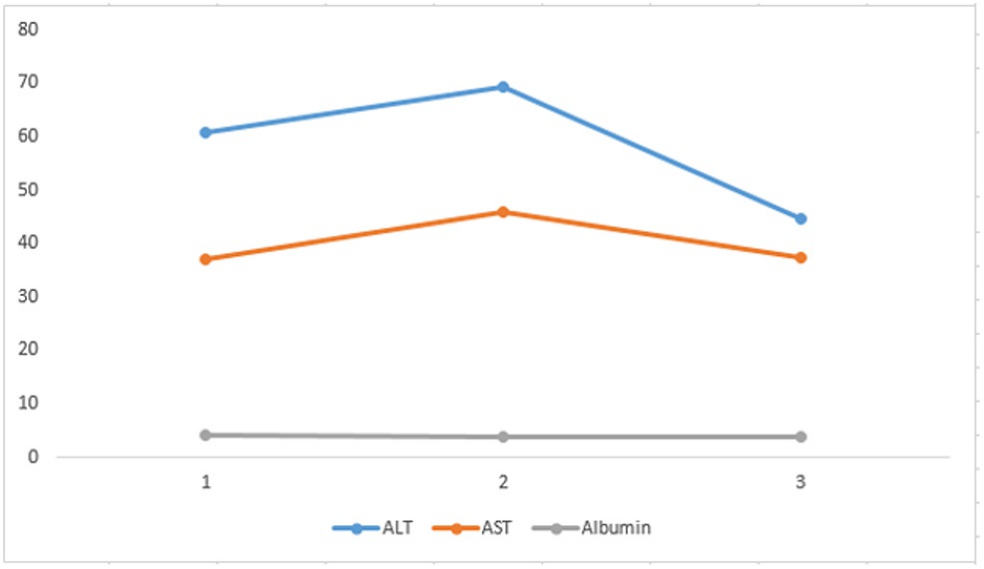
Correlation of ALT, AST, and albumin with viral load in the three groups ALT: alanine transaminase; AST: aspartate aminotransferase

A decreased level of albumin is seen in cases of liver damage and is most likely to be induced by systemic inflammation and adverse drug reactions in critically ill COVID-19 patients. A study reported that a decreased level of albumin correlates with the higher disease severity in critically diseased patients [15]. Similarly, in our study, albumin in all three Ct value-based groups expressed a mean ± standard deviation in its normal range from 3-4 g/dL, showing significance in all the groups' p-value (p<0.05) (Figure 2).

Trends of TLC, Lymphocytes, and Neutrophils

We have found a significance for lymphocytes, neutrophils, and TLC in all the three groups studied (p<0.05). However, there was no significant trend change seen when compared with different groups. A case study conducted by Dr. Chang Zheng Wang analyzed COVID-19 and found that these were normal in the initial phase of the disease. However, with the increase in the progression; however, lymphocyte count decreased. The neutrophil to lymphocyte ratio (NLR) and CRP increased significantly [16].

**Figure 3:**
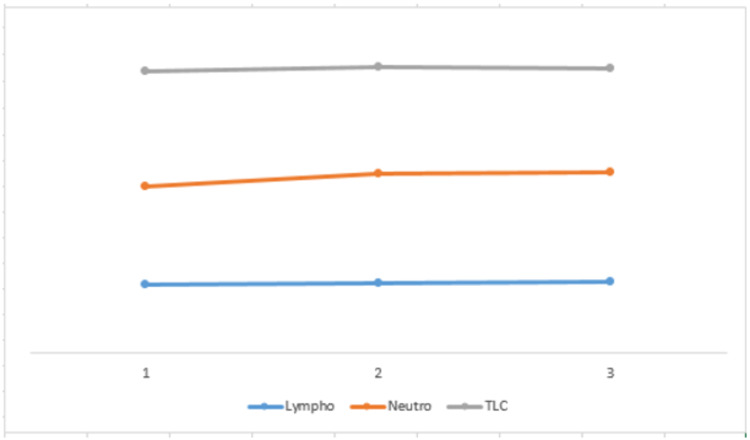
Correlation of lymphocytes, neutrophils, and WBC count with viral load in the three groups

Mardani et al. identified considerably different numbers and percentages of neutrophils (NEU), lymphocytes (LYM), and WBC in COVID-19 RT-PCR positive and negative patients. They found a low number of LYM and WBC in SARS-COV-2-positive patients as compared to the NEU counts, which were elevated in these patients. In former studies, low counts of WBC and LYM have been reported in COVID-19-positive patients [17]. Previous studies reported that COVID-19 acts on the immune cells by inhibiting cellular immune function. SARS-COV-2 propagates through the respiratory tract and involves other cells by stimulating immune responses, which change the number of WBCs, for instance, lymphocytes (Figure 3) [18].

Trends of Ferritin, LDH, and D-Dimer

The biochemical parameter ferritin is involved in the dysregulation of the immune system in the hyperferritinemia condition. Pro-inflammatory and direct immune-suppressive effects result in the production of a cytokine storm. The ramifications of a COVID-19 infection lead to the production of a cytokine storm, and the severity of the disease depends on the cytokine storm syndrome [19]. Raised levels of serum ferritin have been reported in diabetic patients, and it has been identified that they experience serious COVID-19 complications [20].

In a study conducted by Chen et al., the serum ferritin levels of 99 patients were examined, and they found that 63 of them had an elevated serum ferritin range [18]. In this study, ferritin levels were compared with the Ct value. In groups 1, 2, and 3, the p-value was found to be statistically significant for ferritin levels (p< 0.05), which means that COVID-19-positive patients showed higher levels of ferritin in serum.

An increased LDH level indicates a poor prognosis in COVID-19 patients. As LDH (isoenzyme 3) is present in the lungs, severe COVID-19 infected patients may be observed to release a higher level of LDH enzyme in pulmonary circulation, as a critical form of pneumonia, generally emerging into ARDS, which is an indication of disease. Furthermore, the level of LDH is elevated in thrombotic microangiopathy, which is associated with myocardial injury and renal failure [21]. LDH is a major biochemical marker responsible for lung damage and the level of LDH is mostly elevated in COVID-19 patients. The elevated LDH level is responsible for tissue destruction in the lungs, and it is observed as an important biochemical marker in interstitial pulmonary fibrosis [22].

LDH is seen constantly increased in the three (1-3) groups showing a significant p-value (0.05) while in group 1, the p-value was not significant. This parameter didn’t show any trend change with viral load and showed a straight line.

**Figure 4:**
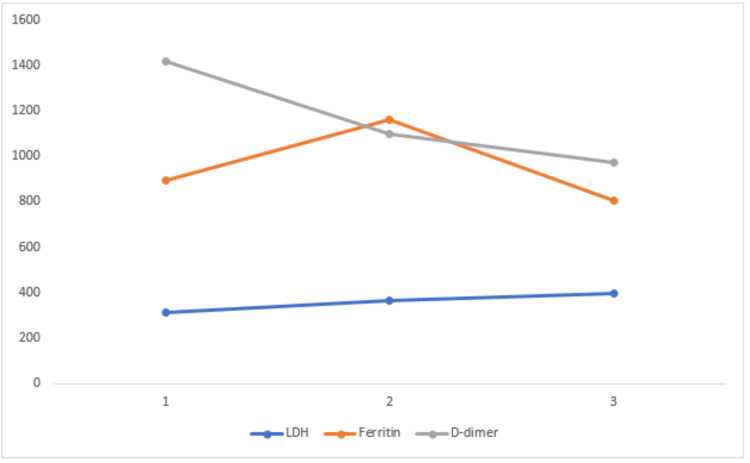
Correlation of biochemical parameters like LDH, ferritin, and D-dimer with viral load in the three groups LDH: lactate dehydrogenase

D-dimer is a product of fibrin breakdown and acts as an indicator of fibrinolytic activity. A correlation between indicators of coagulation activation (D-dimer) and pro-inflammatory cytokines has been observed in COVID-19 critical patients [23]. In a previous study, it has been investigated that D-dimer is dramatically elevated in COVID-19 patients and it helps in diagnosing the disease [24]. The results of the current study also suggest the same, as the D-dimer values were observed to be on the higher side, also showing significance in all the three groups studied (P<0.05). There is a downward trend from 1 to 3 (Figure 4).

Bilirubin

Elevated levels of bilirubin can be due to an increased hemolysis rate or liver damage. Several studies revealed that increased total bilirubin may act as an abnormal diagnostic biochemical parameter in COVID-19 patients [25]. In COVID-19 patients, altered levels of bilirubin have been found, but the tendency of such an alteration in bilirubin level is not clear, especially in relation to disease severity [26].

Bilirubin shows a significant p-value (p<0.05) in all the groups studied, however, no change was observed in their values among the three groups.

Trends of PCT and CRP

Bacterial infection, systemic inflammatory response, and fungal infections contribute to the higher levels of PCT in serum. It has been seen that PCT levels are not raised in case of viral infections generally [27]. The PCT mean was found to be higher in each of these groups but no significance was found in each group, as very few of the total patients included in this study were diagnosed with a critical level of disease severity and, therefore, most were not tested for PCT. In a clinical context, PCT provides insignificant information, as PCT levels can be influenced by comorbid conditions, for instance, congestive heart failure and chronic kidney diseases (CKD) [28].

CRP is a marker for inflammation. It has been observed that CRP is a biochemical parameter that is usually increased in patients with COVID-19. Yang et al. studied 85 COVID 19 patients, in which 96.47% of patients were observed with an increased level of CRP, which is being determined by disease severity [29]. Another study showed that CRP levels were strongly correlated with disease severity and lung lesion in patients with COVID-19 [30].

**Figure 5:**
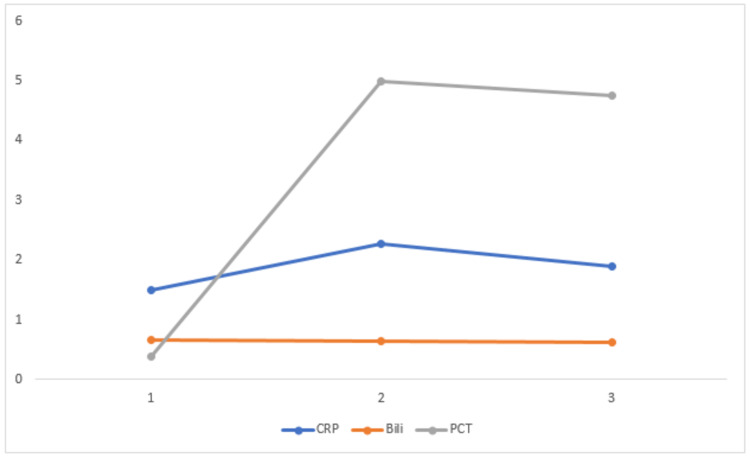
Correlation of biochemical parameters like CRP, bilirubin, and PCT with viral load in three groups PCT: procalcitonin; CRP: c-reactive protein

In this study, the CRP mean ± standard deviation showing increased values in the three groups. CRP levels show significant values (p<0.05) in group 1 (09-20), group 2 (21-30), and group 3 (31-40). There is a downward trend from a higher viral load to a lower viral load group (Figure 5).

Limitations

Complete data of clinical and laboratory parameters were not available for some of the patients. Only nasopharyngeal swabs have been used for the assessment of viral load in patients in different disease severity groups.

Future prospects

More studies should be carried out using other sample sources, i.e., nasopharyngeal and lower respiratory tract samples, such as sputum and/or bronchial lavage, to find out the correlation between the clinical and viral load of SARS-COV-2. 

## Conclusions

The severity of the disease was not statistically significant in the Ct value groups (p>0.05). However, biochemical parameters i.e., ALT, AST, ALP, CRP, and bilirubin, were statistically significant (p<0.05). The patients with COVID-19 should be closely monitored for the assessment of disease progression according to the above-mentioned biochemical parameters.

## References

[1] Abid K, Bari YA, Younas M, Tahir Javaid S, Imran A (2020). Progress of COVID-19 epidemic in Pakistan. Asia Pac J Public Health.

[2] (2020). Pakistan COVID-19 dashboard. http://www.covid.gov.pk/.

[3] Su S, Wong G, Shi W (2016). Epidemiology, genetic recombination, and pathogenesis of coronaviruses. Trends Microbiol.

[4] Rao SN, Manissero D, Steele V, Pareja J (2020). Clinical utility of cycle threshold values in the context of COVID-19 [Preprint]. Research Square.

[5] (2020). WHO. Naming the coronavirus disease (COVID-19) and the virus that causes it. https://www.who.int/emergencies/diseases/novel-coronavirus-2019/technical-guidance/naming-the-coronavirus-disease-(covid-2019)-and-the-virus-that-causes-it.

[6] Das B, Bhatia SY, Pal PM (2021). Evaluation of the role of routine laboratory biomarkers in COVID-19 patients: perspective from a tertiary care hospital in India. Indian J Clin Biochem.

[7] Rao SN, Manissero D, Steele VR, Pareja J (2020). A systematic review of the clinical utility of cycle threshold values in the context of COVID-19. Infect Dis Ther.

[8] Yu F, Yan L, Wang N (2020). Quantitative detection and viral load analysis of SARS-CoV-2 in infected patients. Clin Infect Dis.

[9] Letícia de Oliveira Toledo S, Sousa Nogueira L, das Graças Carvalho M, Romana Alves Rios D, de Barros Pinheiro M (2020). COVID-19: review and hematologic impact. Clin Chim Acta.

[10] Aquino-Jarquin G (2021). The raw cycle threshold values from reverse-transcription polymerase chain reaction detection are not viral load quantitation units. Clin Infect Dis.

[11] Rabaan AA, Tirupathi R, Sule AA (2021). Viral dynamics and real-time RT-PCR Ct values correlation with disease severity in COVID-19. Diagnostics (Basel).

[12] Mardani R, Vasmehjani AA, Zali F (2020). Laboratory parameters in detection of COVID-19 patients with positive RT-PCR; a diagnostic accuracy study. Archi Acad Emerg Med.

[13] Zhang C, Shi L, Wang FS (2020). Liver injury in COVID-19: management and challenges. Lancet Gastroenterol Hepatol.

[14] Wang Q, Zhao H, Liu L (2020). Characteristics and change patterns of liver function in 105 hospitalized adults patients with COVID-19 in Beijing, China [Preprint]. Research Square.

[15] Li J, Li M, Zheng S (2020). Plasma albumin levels predict risk for nonsurvivors in critically ill patients with COVID-19. Biomark Med.

[16] (2020). Case study: CBC&CRP results of a critically ill COVID-19 patient. https://www.mindray.com/en/presscenter/Case_Study__CBC_CRP_results_of_a_critically_ill_COVID-19_patient.html.

[17] Qu R, Ling Y, Zhang YH (2020). Platelet-to-lymphocyte ratio is associated with prognosis in patients with coronavirus disease-19. J Med Virol.

[18] Chen N, Zhou M, Dong X (2020). Epidemiological and clinical characteristics of 99 cases of 2019 novel coronavirus pneumonia in Wuhan, China: a descriptive study. Lancet.

[19] Huang C, Wang Y, Li X (2020). Clinical features of patients infected with 2019 novel coronavirus in Wuhan, China. Lancet.

[20] Son NE (2019). Influence of ferritin levels and inflammatory markers on HbA1c in the type 2 diabetes mellitus patients. Inflammatory markers on HbA1c in the type 2 diabetics. Pak J Med Sci.

[21] Rodriguez-Morales AJ, Cardona-Ospina JA, Gutiérrez-Ocampo E (2020). Clinical, laboratory and imaging features of COVID-19: a systematic review and meta-analysis. Travel Med Infect Dis.

[22] Yan L, Zhang H-T, Goncalves J (2020). An interpretable mortality prediction model for COVID-19 patients. Nat Mach Intell.

[23] Pettilä V, Hynninen M, Takkunen O, Kuusela P, Valtonen M (2002). Predictive value of procalcitonin and interleukin 6 in critically ill patients with suspected sepsis. Intensive Care Med.

[24] Zhang L, Yan X, Fan Q, Liu H, Liu X, Liu Z, Zhang Z (2020). D-dimer levels on admission to predict in-hospital mortality in patients with Covid-19. J Thromb Haemost.

[25] Kaplan B, Meier-Kriesche HU (2002). Death after graft loss: an important late study endpoint in kidney transplantation. Am J Transplant.

[26] Paliogiannis P, Zinellu A (2020). Bilirubin levels in patients with mild and severe Covid-19: a pooled analysis. Liver Int.

[27] Albrich WC, Harbarth S (2015). Pros and cons of using biomarkers versus clinical decisions in start and stop decisions for antibiotics in the critical care setting. Intensive Care Med.

[28] Yunus I, Fasih A, Wang Y (2018). The use of procalcitonin in the determination of severity of sepsis, patient outcomes and infection characteristics. PLoS One.

[29] Yang W, Cao Q, Qin L (2020). Clinical characteristics and imaging manifestations of the 2019 novel coronavirus disease (COVID-19): a multi-center study in Wenzhou city, Zhejiang, China. J Infect.

[30] Warusevitane A, Karunatilake D, Sim J, Smith C, Roffe C (2016). Early diagnosis of pneumonia in severe stroke: clinical features and the diagnostic role of C-reactive protein. PLoS One.

